# Bis-phenylethynyl polyhedral oligomeric silsesquioxanes: new high-temperature, processable thermosetting materials†

**DOI:** 10.1039/c8ra05954c

**Published:** 2018-07-31

**Authors:** Levi M. J. Moore, Jacob J. Zavala, Jason T. Lamb, Josiah T. Reams, Gregory R. Yandek, Andrew J. Guenthner, Timothy S. Haddad, Kamran B. Ghiassi

**Affiliations:** Air Force Research Laboratory, Aerospace Systems Directorate, Edwards AFB California 93524 USA levi.moore.1@us.af.mil kamran.ghiassi@us.af.mil; ERC Incorporated, Edwards AFB California 93524 USA; Nano Hydrophobics, Inc. San Francisco CA 94115 USA andrewguenthner@gmail.com

## Abstract

Bis-phenylethynyl polyhedral oligomeric silsesquioxane (bis-PE-POSS) compounds were synthesized and thermally cured yielding crosslinked materials. After curing at 370 °C, thermal decomposition occurs near 600 °C under nitrogen. These materials were synthesized by condensation of a new phenylethynyl-functional dichlorosilane onto tetrasilanol phenyl POSS, yielding two geometric isomers.

## Abstract 2

After thermal crosslinking, bis-phenylethynyl POSS exhibits 5% mass loss temperatures approaching 600 °C, the highest of reported POSS compounds.

## Graphical abstract

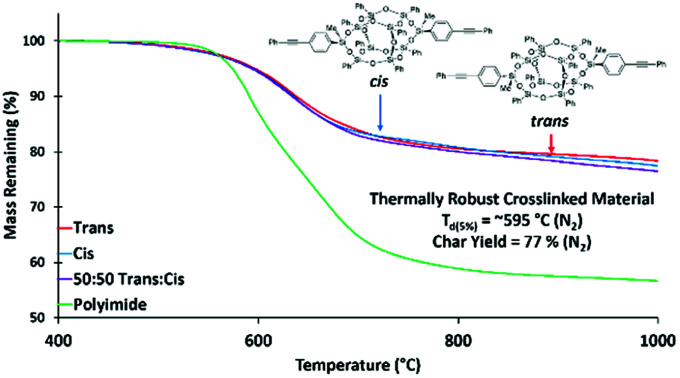

Various composite applications, especially in aerospace, call for polymeric matrix materials with high glass transition temperatures (*T*_g_s) and good thermo-oxidative stability, able to withstand exposure to temperatures in excess of 300 °C while maintaining their mechanical robustness.^1^ A continuous service temperature of 370 °C is widely recognized as a very important technical challenge for organic matrix composites.^2^ Some thermosetting systems, such as polycyanurates,^3,4^ poly(benzoxazines),^5^ and phenylethynyl-endcapped poly(oligoimides)^6^ have approached that benchmark, but few resins possess the requisite combination of processability, mechanical properties, and thermo-oxidative stability. Those that can be utilized in service over 300 °C have finite lifetimes, especially in the presence of oxidizing species, and exhibit poor processability, requiring solvents for fiber impregnation and high pressures to promote flow. A matrix material that shows a significant improvement in thermochemical stability at high temperatures with an expanded processing window without the utilization of solvents would be a major step forward in the pursuit of next-generation resins.

One of the first examples of advancement towards this goal is NASA's PETI (phenylethynyl-terminated imide) series of phenylethynyl endcapped poly(oligoimide) resins.^7–9^ They exhibit high glass transition temperatures, excellent solvent resistance, and good mechanical properties, but have relatively poor flow characteristics, often requiring pressure during implementation of complex curing cycles. The robustness of the material can be attributed to the rigidity afforded by the many imide groups in the polymer, as well as phenylethynyl (PE) crosslinking chemistry, yielding a material with a broad processing window and good thermo-oxidative stability.

Common in high temperature thermosets, phenylethynyl crosslinking chemistry is a versatile route for the production of a variety of thermosetting materials. The robustness of this chemistry comes from the apparent cyclization of three phenylethynyl groups to form hexasubstituted benzene rings as the crosslink junctions in the final product, as well as higher order rings and other ill-defined but still olefinic structures.^6,10^ The properties of the final material are highly modular, with the crosslink density and molecular weight between crosslinks readily tunable through the size of the oligomer or polymer between the phenylethynyl groups.^9^

Also of note for their thermo-oxidative stability, polyhedral oligomeric silsesquioxane (POSS) compounds are hybrid organic–inorganic molecules composed of an inorganic silicon oxide core with an organic corona, well studied and available with a variety of functional groups. Phenyl-substituted POSS compounds are the most thermally stable in their class, due to their high aromatic content as well as the inorganic silsesquioxane core.^11^ Phenyl POSS compounds have found utility as additives for viscosity modification in high-temperature polymers, imparting improved processability and thermal properties when blended into the neat polymer, with little to no sacrifice in mechanical properties.^12,13^

There have been many efforts to covalently attach POSS to polymeric materials as well,^14^ including into polyimides as pendent moieties^15,16^ or even as monomers in the backbone of the polymer.^17,18^ This sort of incorporation increases the service lifetime of the fabricated part by reducing the amount of wear experienced. When exposed to low-Earth-orbit, Kapton-like polyimides with POSS moieties incorporated into the main chain of the polymer experienced erosion much more slowly than that of the neat polyimide due to the formation of a passivating silica layer.^19^ Additionally, phenylethynyl-functional POSS polyimide oligomers were found to be readily crosslinkable through the standard phenylethynyl cure schedule.^20^ These POSS-containing thermosets showed reduced moisture uptake when compared to the control polyimide, accomplished by the inclusion of the hydrophobic silsesquioxane cage of the POSS moieties, although with a reduction in the *T*_g_ of the finished material compared to the control. A strategy was developed to design a new matrix material that combined the high temperature properties of the POSS and phenylethynyl groups without any adverse effects of moisture uptake by removing the intermediate imide linkages. Here we present a new material, bis-PE-POSS, which utilizes the hybrid POSS architecture along with phenylethynyl curing chemistry. We report the synthetic route to this compound and demonstrate its exceptional thermal stability, with 5% mass loss temperatures approaching 600 °C.

POSS compounds are highly modular, and are commercially available with a variety of organic corona functionalities and reactive groups. This report builds on the library of functionalities available for condensation onto open POSS cages, yielding a compound with exceptional thermal stability. This synthetic procedure was modified from our previous report on the synthesis of aromatic POSS dianilines^21,22^ by the preparation of the phenylethynyl dichlorosilane (Scheme 1), yielding thermally robust compounds with a largely aromatic organic corona. Phenylethynyl bromide is reacted with magnesium turnings in THF to yield the Grignard reagent (1) as an emerald green solution which, after filtration to remove the unreacted magnesium, is added dropwise to excess methyltrichlorosilane in THF to yield the phenylethynyl methyl dichlorosilane (2). This dichlorosilane is a pale yellow solid and is easily purified through sublimation or recrystallization from hexanes, yielding pure product as seen by ^29^Si NMR (detailed synthetic procedures, NMR, and FTIR spectra are provided in the ESI†).

**Scheme 1:**
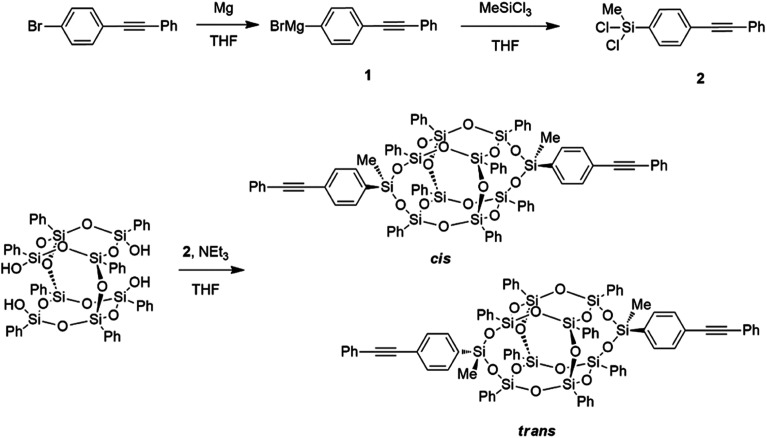
Synthetic route to bis-PE POSS.

To condense the phenylethynyl dichlorosilane onto the POSS cage, the phenyl tetrasilanol POSS was first dissolved in THF, and a THF solution of triethylamine and dichlorosilane, both at a slight excess with respect to the silanol functionality, was added dropwise. Due to the asymmetry of the dichlorosilane, with a methyl group and a phenylethynyl group attached to the silicon, there are two different conformations that the resulting condensed cages can adopt, termed *cis* and *trans*. The *cis* isomer has both phenylethynyl groups arranged pointing the same way, and the *trans* has them both pointing opposite each other, leading to a more crystalline material that readily packs.

This subtle variation in crystal packing led to a difference in solubility between the two isomers. A solid precipitate formed in the reaction mixture after the condensation step that contained both triethylamine hydrochloride and the *trans*-bis-phenylethynyl POSS. After filtration of the reaction mixture, the filter cake was washed with methanol, and then stirred in methanol to remove any remaining salt, yielding pure *trans*-bis-phenylethynyl POSS. Less-crystalline *cis*-bis-phenylethynyl-POSS cages remained in solution, along with some *trans*-isomer. The filtrate was reduced in volume and precipitated into methanol. The solids were removed by filtration, which yielded a mixture of *cis*- and *trans*-isomers. Pure *cis*-isomer was obtained by selective extraction of the mixture with diethyl ether, in which the *trans* isomer is only sparingly soluble. Hansen solubility parameters were calculated based on group contribution models for POSS compounds,^23^ and despite the isomers' significant solubility differences, both compounds have estimated values of *δ*_D_ = 19.4 ± 0.6, *δ*_P_ = 6.9 ± 2.0, and *δ*_H_ = 5.7 ± 1.6. Estimated “radius of interaction” values (for minimal solubility) of the isomers, though, are around 12–16 for the *cis* compound compared to only 6–10 for the *trans* compound (see ESI†). After isolation of the two different compounds, mixtures of *cis*- and *trans*-isomers were then formulated to determine the effect of stereochemistry on thermal properties.

The ^29^Si NMR spectra of the bis-phenylethynyl-POSS isomers are shown in Fig. 1. The full spectrum (a) shows a mixture of both isomers, as does (b), which along with the *cis* isomer in (c) and *trans* isomer in (d) are zoomed in to the regions of interest. Sharp peaks demonstrate that the POSS cages retain their discrete molecular structure through the functionalization step. The chemical shift from the silicon atoms that tether the phenylethynyl groups to the POSS cage is evident at −31.1 ppm (highlighted in blue), and the shifts from the two silicon atoms that are attached to the tethering silicon (through oxygens) are evident at −78.2 ppm (red). The central silicon atoms, then, show different shift patterns depending on the isomer. Because of the high symmetry of the *trans* isomer, the central silicon atoms exhibit only one peak (purple). The *cis* isomer's central silicon atoms are in differing chemical environments, with the shifts from the two atoms opposite the phenylethynyl groups at −79.6 ppm (green), and the shifts from the atoms between the phenylethynyl groups appearing at −79.1 ppm (orange).^24^

**Fig. 1:**
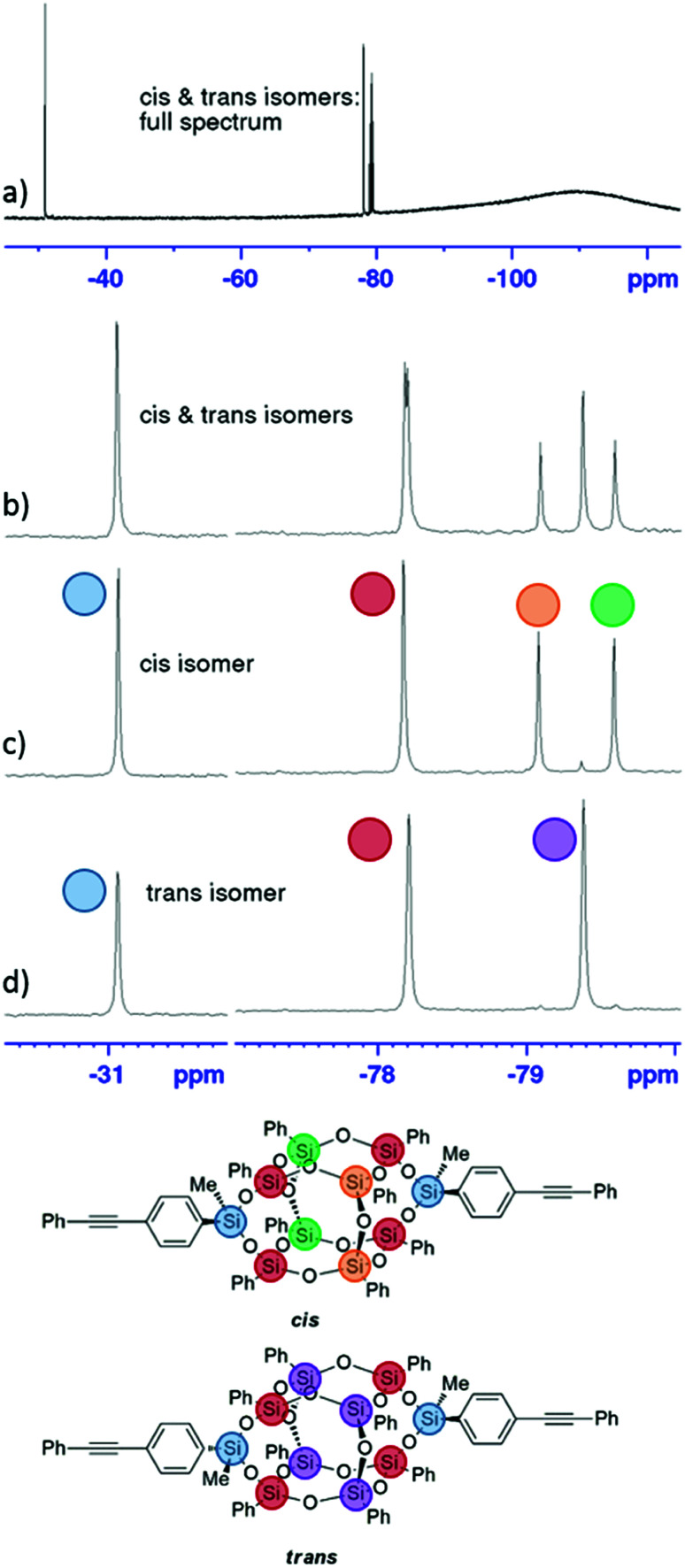
^29^Si NMR spectra of bis-PE POSS isomers. The full spectrum of a mixture of isomers is shown in (a), and zoomed in to the regions of interest in (b). The *cis-*isomer is shown in (c), and the *trans-*isomer in (d). Residual *trans*-isomer is evident in the spectrum for the *cis*-isomer (c).

Single crystals suitable for X-ray diffraction were grown from concentrated solutions of benzene containing either *cis* or *trans* bis-PE-POSS. The crystal structures, presented in Fig. 2, clearly show the different orientations between the regioisomers. While both structures are found in the same space group (triclinic, *P*1̄), the *cis* isomer is found on a crystallographic general position, while the *trans* isomer resides on a center of inversion. Interestingly, the structures suggest that the *cis* isomer belongs to the *C*_2v_ point group while the *trans* is *C*_2h_.

**Fig. 2:**
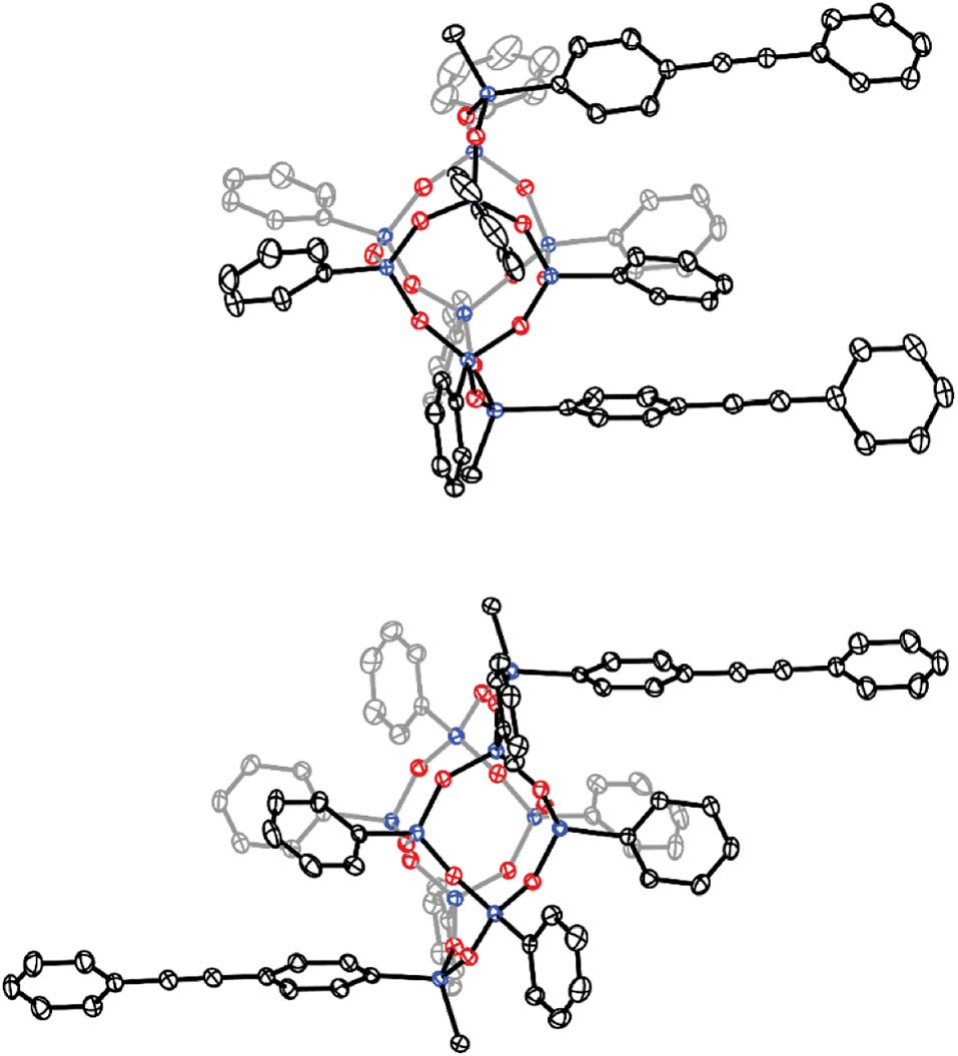
X-ray crystal structures for *cis* (top) and *trans* (bottom) regioisomers of the bis-PE POSS compounds. Thermal ellipsoids are plotted at 30%. Colors represent silicon (blue), oxygen (red) and carbon (black). Hydrogen positions and solvate molecules are omitted for clarity.

Thermal studies were performed in order to ascertain the processability and thermal properties of the bis-PE-POSS compounds. Differential scanning calorimetry (DSC) showed distinct melting temperatures for the two isomers (Fig. 3a). Melting endotherms were observed for both isomers, with the *cis*-isomer showing a melting transition at 261 °C and the *trans*-isomer at 304 °C. The *cis*-isomer's lower crystallinity led to its lower melting point. The blend of the isomers exhibits two endotherms as expected. The *cis*-isomer showed no change in melting point in the mixture, but the *trans*-isomer saw a depression in melting point to 280 °C, presumably from solubilization into the *cis*-isomer. The compounds are free-flowing thin liquids in the melt state before cure. Both isomers and mixtures of isomers showed a cure exotherm with a maximum temperature around 379 °C. A representative exotherm is shown in Fig. 3b.

**Fig. 3:**
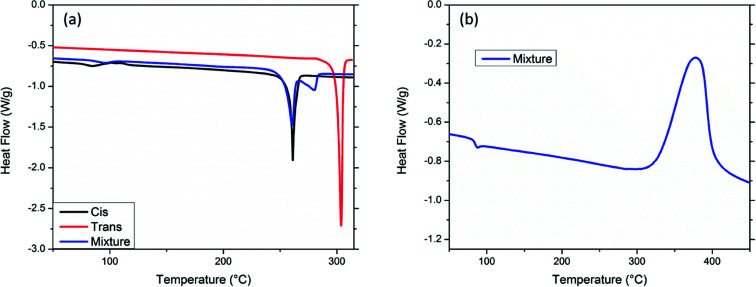
Differential scanning calorimetry curves (exo up) for *cis*, *trans*, and a 50 : 50 mixture of bis-PE-POSS showing melting points (a). Representative cure exotherm for a 50 : 50 mixture of bis-PE-POSS isomers (b).

Thermogravimetric analysis shows the exceptional thermal stability of these compounds. TGA curves are shown in Fig. 4, and detailed thermal properties are shown in Table 1. The phenylethynyl groups on the POSS cage crosslink and cure above 320 °C with a maximum rate at 370 °C, leading to a highly crosslinked system with increased aromatic character compared to the discrete molecules themselves. The *cis* isomer, *trans* isomer, or a 50/50 mixture of the two were cured *in situ* in a TGA pan with an isothermal hold at 370 °C for 1 h. The resulting cured bis-PE-POSS resin was then subjected to a ramp to 1000 °C at a rate of 10 °C min^−1^. In a nitrogen atmosphere, 5% mass loss occurred around 595 °C for each isomer, as well as the mixture of isomers. The char yields are also quite high, all around 77%. For comparison, a known phenylethynyl-endcapped polyimide, 6-FDA-ODA-PEPA,^20^ was subjected to the same thermal treatment, and shows a 5% mass loss temperature of 574 °C and a char yield of 57%. Analysis in air shows quite high degradation temperatures as well. The polyimide left no char yield after ramping to 1000 °C, but the char yields of the bis-PE POSS compounds in air were 43% for the *trans* isomer, 36% for the *cis* isomer, and 39% for the mixture, all close to the theoretical value for the mass fraction of the central inorganic cage. The carbonaceous content of the cured resin was oxidized and lost, leaving behind the nonvolatile silicon oxide portions of the resin.

**Fig. 4:**
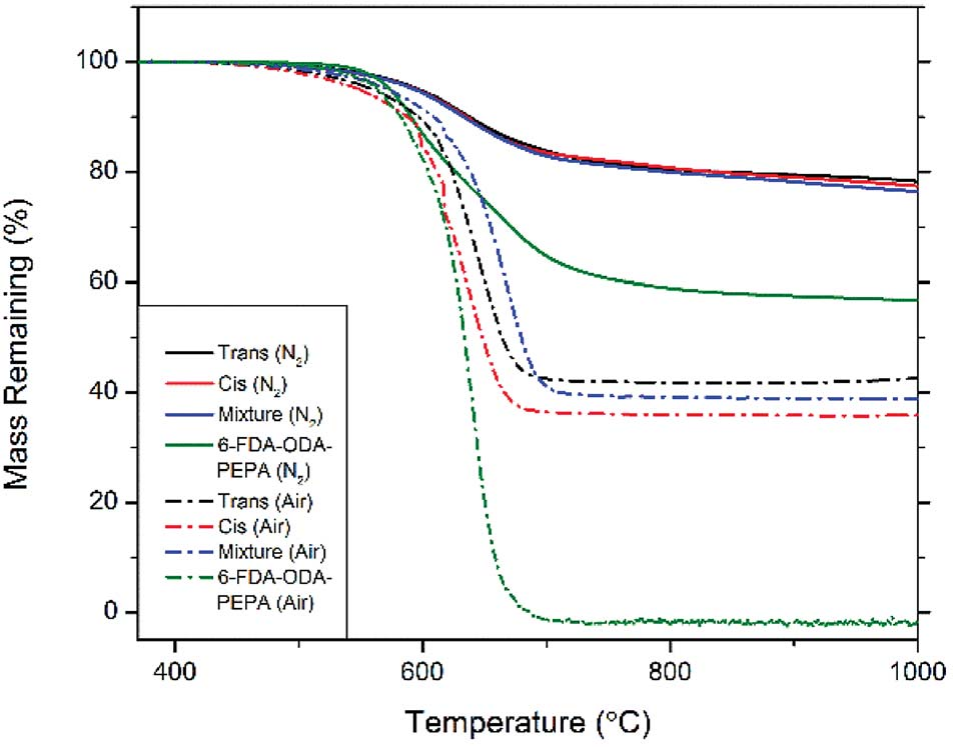
TGA curves of bis-PE-POSS in nitrogen (solid lines) and air (dashed lines).

**Table 1:** Thermal properties of *in situ* cured bis-PE-POSS resin

Material	Atmosphere	5% loss *T* (°C)	Char yield (%)
*trans*-bis-PE-POSS	N_2_	596	78
	Air	559	43
*cis*-bis-PE-POSS	N_2_	594	77
	Air	548	36
50 : 50 *trans* : *cis*-bis-PE-POSS	N_2_	592	76
	Air	571	39
6-FDA-ODA-PEPA	N_2_	574	57
	Air	564	0

These compounds show an increase in degradation temperature as compared to other phenyl substituted POSS compounds as well. Octaphenyl POSS has a degradation and volatilization temperature around 450 °C and char yield of 70% in nitrogen,^11,25^ well below that of the bis-PE-POSS. The crosslinking and increase in aromatic content in the bis-PE POSS compounds improves thermal stability compared to other POSS systems, leading to a more robust material.

## Conclusions

In conclusion, we have synthesized a phenylethynyl-functional POSS compound through a modular synthesis that shows exceptional thermal stability to temperatures approaching 600 °C. This compound is able to be utilized as a thermoset resin, and is crosslinked through cyclization of its phenylethynyl groups. This crosslinking process greatly enhances its thermal stability in comparison to high temperature polymers, and even improves on properties seen in POSS compounds with similar functionality through inclusion of the curable phenylethynyl group. This compound opens up the possibility of thermoset resins with ever greater thermal stabilities and operational temperatures.

## Conflicts of interest

There are no conflicts to declare.

## Supplementary Material

RA-008-C8RA05954C-s001

RA-008-C8RA05954C-s002
