# Supplementary material for: Bis-phenylethynyl polyhedral oligomeric silsesquioxanes: new high-temperature, processable thermosetting materials
Source: RSC Adv. 2018 Jul 31;8(48):27400–5. doi: 10.1039/c8ra05954c (PMC9083338; doi:10.1039/c8ra05954c)
Supplement: RA-008-C8RA05954C-s001 [file RA-008-C8RA05954C-s001.pdf]

Electronic Supplementary Material (ESI) for RSC Advances.
This journal is © The Royal Society of Chemistry 2018

Bis-phenylethynyl Polyhedral Oligomeric Silsesquioxanes: New High-Temperature, Processable Thermosetting Materials

Levi M. J. Moore,[a*] Jacob J. Zavala,[b] Jason T. Lamb,[b] Josiah T. Reams,[b] Gregory R. Yandek,[a] Andrew J. Guenthner,[a,c*] Timothy S. Haddad,[b] Kamran B. Ghiassi[a*]

[a] Air Force Research Laboratory, Aerospace Systems Directorate, Edwards AFB, California 93524, United States

[b] ERC Incorporated, Edwards AFB, California 93524, United States

[c] Nano Hydrophobics, Inc., San Francisco, CA 94115, United States

Table of Contents

1. **Materials and General Considerations**
2. **Synthesis**
3. **NMR**
4. **FTIR**
5. **Single crystal X-ray diffraction**
6. **Thermal Analysis**
7. **Estimation of Hansen Solubility Parameters (HSP)**

1. Materials and General Considerations

All solvents were obtained commercially at a purity of 95% or greater and used as received unless specified otherwise. All reagents were used as received unless specified otherwise. Magnesium turnings (98%) were obtained from Aldrich, and methyl trichlorosilane (99%) was obtained from Aldrich and distilled under dry nitrogen prior to use. Triethylamine was obtained from Aldrich and distilled from calcium hydride prior to use. 4-bromo-1-phenylethynylbenzene was obtained from TCI America. Tetrasilanol phenyl POSS was obtained from Hybrid Plastics. $CDCl_3$ for NMR analysis was obtained from Cambridge Isotopes and dried over molecular sieves. NMR spectra were obtained on a Bruker Avance III HD 400 MHz spectrometer using $CDCl_3$ as the solvent, and chemical shifts referenced with respect to solvent (7.26 ppm for ^{1}H and 77 ppm for ^{13}C). ^{29}Si spectra were referenced to external tetramethylsiane at 0 ppm. FTIR data were collected on Nicolet iS50 FTIR spectrometer equipped with an ATR accessory. Elemental analysis was conducted by Atlantic Microlab, Inc.

2. Synthesis

Phenylethynylphenyl methyl dichlorosilane (Cl_2MeSi-PE)

In a nitrogen-filled glovebox, magnesium turnings (2.599 g, 0.107 mol, 1.1 eq) were added to a 250 mL round bottom flask with a magnetic stirrer, and suspended in 50 mL dry THF. 4-bromo-1-phenylethynylbenzene (PE-Br) (25 g, 0.097 mol, 1 eq) was dissolved in 50 mL dry THF, and the solution was added to an addition funnel. The PE-Br solution was added to the magnesium suspension, forming the Grignard reagent as an emerald-green solution. The reaction is quite exothermic, so care was taken to add the PE-Br slowly to avoid boiling the solution. Upon addition of all PE-Br, the solution of Grignard reagent is roughly 1 M in concentration.

After the reaction cooled to room temperature, the solution was filtered through a cotton plug in a glass funnel back into the same addition funnel, and the round bottom and cotton plug were rinsed with a small amount of THF. Methyl trichlorosilane (29.1 g, 0.195 mol, 2 eq) was added to a 500 mL round bottom flask and dissolved in 50 mL THF. The Grignard reagent was added dropwise to the methyl trichlorosilane solution, turning brown upon addition. The solution was left to stir overnight, and the color lightened to a light brown the next morning.

The round bottom flask was equipped with a 180° adapter, removed from the glovebox, and the THF and excess methyltrichlorosilane were removed under dynamic vacuum. Upon removal of the THF, MgClBr salts precipitated out of solution. After the solids formed, the flask remained under dynamic vacuum for 4 hours to remove all volatiles. After this, the flask was transferred back into the glovebox and hexanes (250 mL) were added to dissolve the phenylethynylphenyl methyl dichlorosilane, and left to stir overnight. The salt was filtered off, yielding a yellow Cl_2MeSi-PE solution. The flask was again removed from the glovebox and the majority of the hexanes was removed via dynamic vacuum, until Cl_2MeSi-PE precipitated from solution. The solution was heated with a heat gun on low to re-dissolve the compound, and left to crystallize overnight. The product was filtered off, yielding a pale yellow powder. The mother liquor was reduced in volume via dynamic vacuum, and a second batch of product was recovered from the solution. In total, 18.23 g Cl_2MeSi-PE was recovered (64.4% yield) and characterized via ^1H, ^{13}C, and ^{29}Si NMR spectroscopy. FTIR and EA were not performed due to the air sensitivity of the sample. ^1H NMR (400 MHz, $CDCl_3$): δ 7.73 – 7.57 (m, 6H), 7.38, 7.37 (m, 3H), 1.06 (s, 3H). ^{13}C NMR (101 MHz, $CDCl_3$): δ 133.2, 133.0, 131.7, 131.2, 128.7, 128.4, 126.7, 122.8, 91.6, 88.7, 5.5. ^{29}Si NMR (79 MHz, $CDCl_3$): δ 18.4.

bis-Phenylethynyl POSS (bis-PE POSS)

Tetrasilanolphenyl-POSS (15.00 g, 14.03 mmol, 1 eq) was added to a 500 mL round bottom flask equipped with a magnetic stirbar, and dissolved in a minimal amount of dry THF. Cl_2MeSi-PE (8.580 g, 29.45 mmol, 2.1 eq) was dissolved in dry THF, as was triethylamine (5.960 g, 58.90 mmol, 4.2 eq), and both solutions combined. The Cl_2MeSi-PE/triethylamine solution was added dropwise to the tetrasilanol solution, where a white precipitate formed, and left to stir overnight. The solids were filtered off, yielding a white solid that contained triethylamine hydrochloride and the *trans*- isomer of di-PEPOSS. The filter cake was suspended in methanol to dissolve the salt, filtered, suspended again in fresh methanol, and left to stir for 24 hr. The solution was filtered and yielded 8.03 g of pure *trans*-isomer. The THF solution was reduced in volume until the solids started to precipitate from solution, forming a slurry, and added dropwise to vigorously stirred methanol. The suspension was stirred for 24 hr and filtered, yielding 12.44 g of a mixture of *cis*- and *trans*- isomers. Together, the reaction yielded 20.47 g bis-PE POSS (96.9 % yield), and products were characterized via ^{1}H, ^{13}C, ^{29}Si NMR, and FTIR spectroscopy in addition to single-crystal X-ray diffraction and elemental analysis.

cis isomer: ^{1}H NMR ($CDCl_3$, 400 MHz) δ 7.65 – 7.10 (m, 58H), 0.55 (s, 6H). ^{13}C NMR (101 MHz, $CDCl_3$): *δ* 136.4, 134.1, 134.0, 133.9, 133.4, 131.7, 131.6, 131.0, 130.9, 130.5, 130.3, 128.4, 127.8, 127.7, 127.5, 124.8, 123.3, 90.2, 89.4, -0.58. ^{29}Si NMR (79 MHz, $CDCl_3$): *δ* -31.1, -78.2, -79.1, -79.7. FTIR-ATR: 3072, 3051, 3020, 2965, 1961, 1890, 1822, 1777, 1666, 1594, 1570, 1536, 1506, 1487, 1443, 1430, 1394, 1383, 1307, 1265, 1192. 1078, 1028, 1019, 998, 916, 847, 827, 815, 798, 768, 757, 728, 740, 716, 685, 616, 628, 595, 574, 545. EA calc'd (%) for cis-(bis-PE)-POSS $C_{78}H_{64}Si_{10}O_{14}$: C, 62.20 %; H, 4.28. Found: C, 62.26; H, 4.34.

trans isomer: ^{1}H NMR ($CDCl_3$, 400 MHz) δ 7.66 – 7.20 (m, 58H), 0.56 (s, 6H). ^{13}C NMR (101 MHz, $CDCl_3$): *δ* 136.4, 134.0, 133.9, 133.4, 131.7, 131.6, 130.9, 130.6, 130.5, 130.4, 128.4, 127.8, 127.6, 124.8, 123.2, 90.2, 89.4, -0.56. ^{29}Si NMR (79 MHz, $CDCl_3$): *δ* -31.1, -78.2, -79.4. FTIR-ATR: 3071, 3051, 3026, 2161, 2036, 1979, 1961, 1891, 1824, 1668, 1595, 1571, 1535, 1505, 1487, 1442, 1430, 1393, 1382, 1307, 1267, 1194, 1130, 1088, 1029, 1019, 998, 974, 955, 923, 914, 844, 826, 814, 771, 760, 742, 717, 697, 641, 623, 612, 574, 545. EA calc'd (%) for *trans*-(bis-PE)-POSS $C_{78}H_{64}Si_{10}O_{14}$: C, 62.20 %; H, 4.28. Found: C, 62.27; H, 4.28.

3. Nuclear Magnetic Resonance Spectroscopy

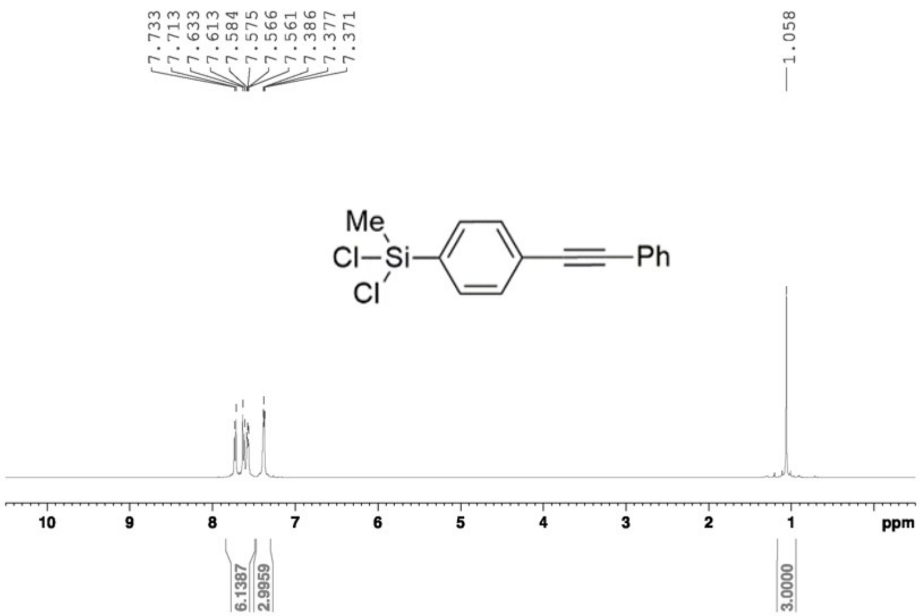

Figure S1. ^{1}H NMR Spectrum of dichlorosilane **2**.

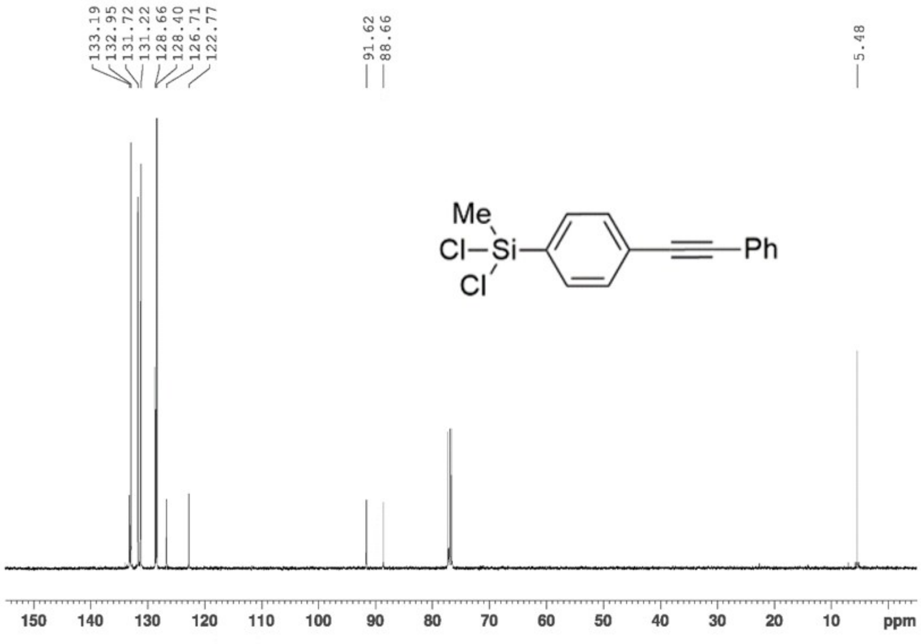

Figure S2. ^{13}C NMR Spectrum of dichlorosilane **2**.

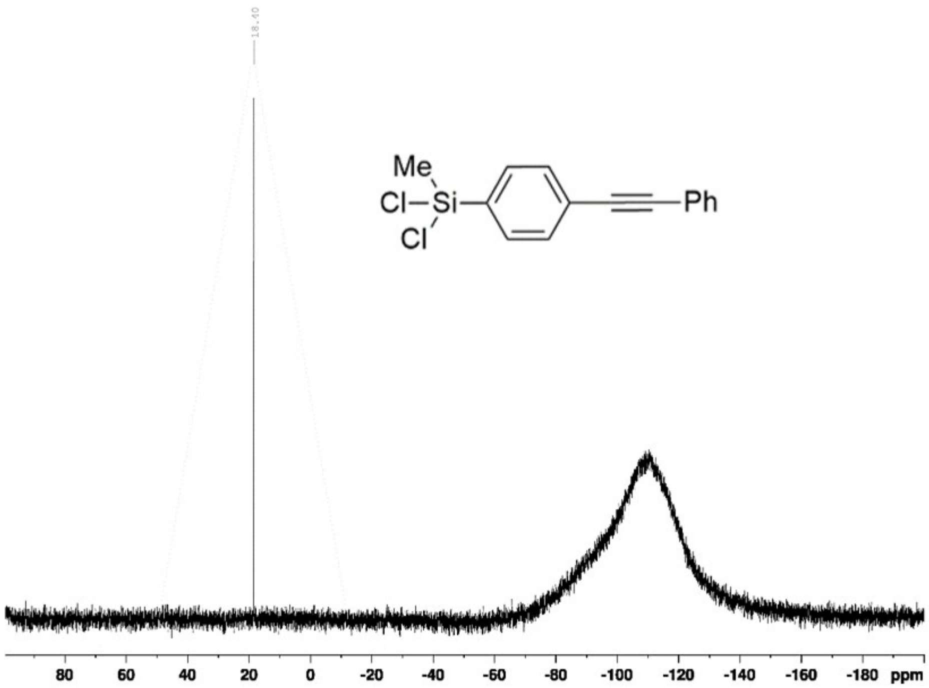

Figure S3. ^{29}Si NMR Spectrum of dichlorosilane **2**.

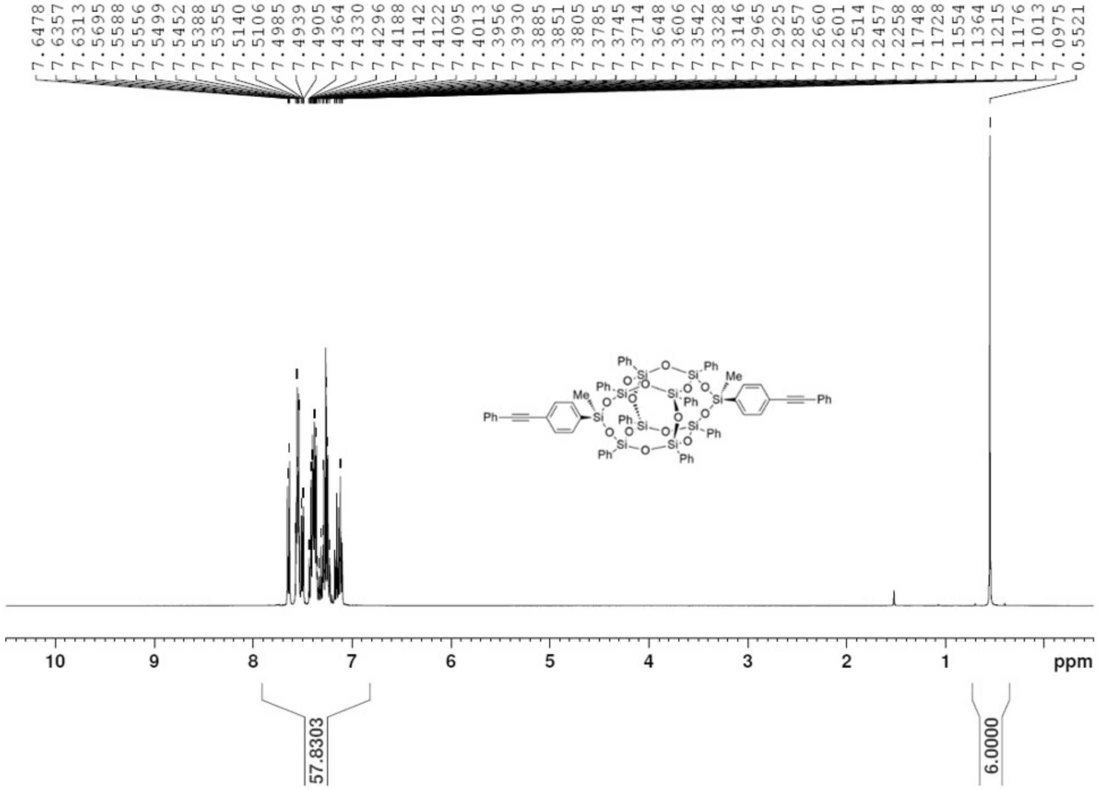

Figure S4. ^{1}H NMR spectrum of bis-(cis-PE)-POSS

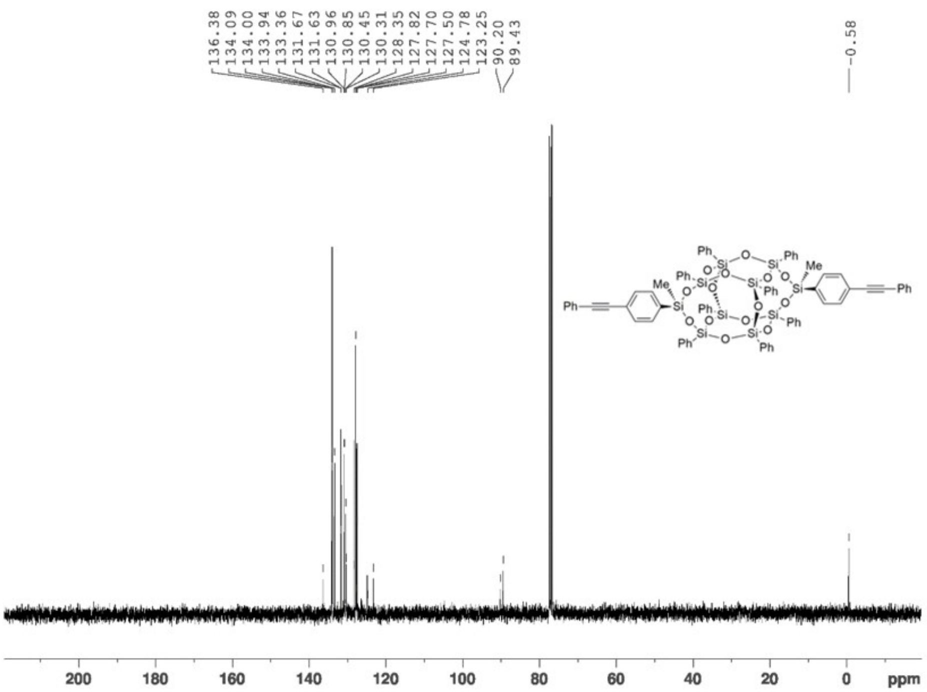

Figure S5. ^{13}C NMR spectrum of bis-(cis-PE)-POSS

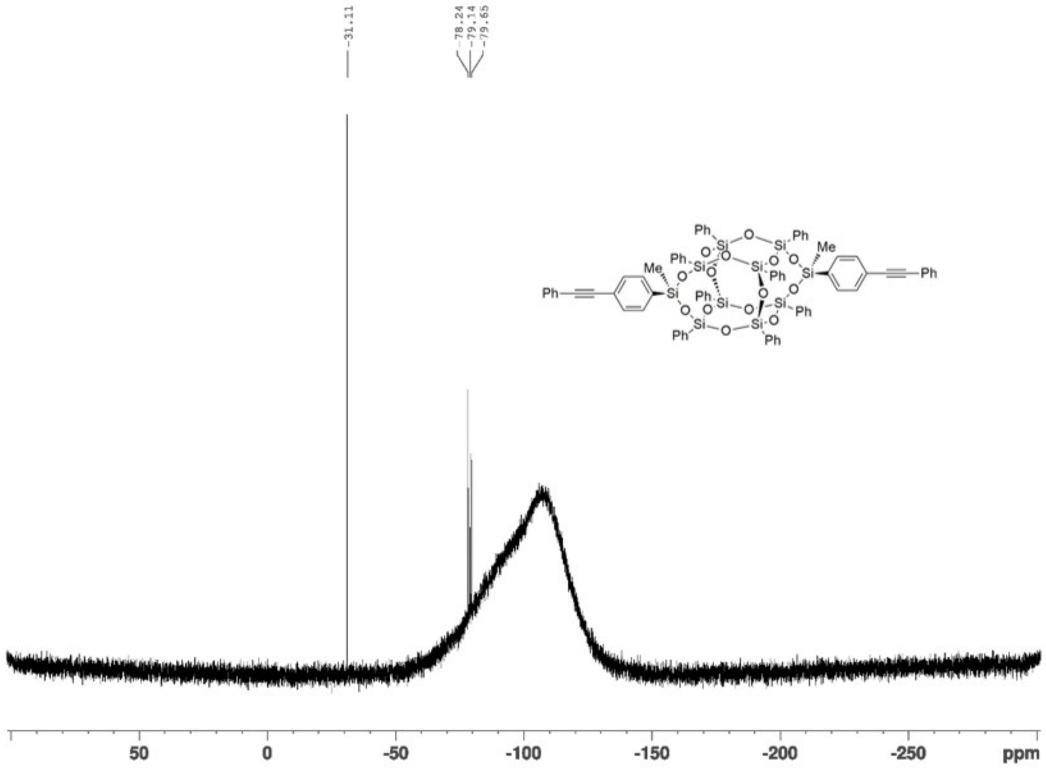

Figure S6. ^{29}Si NMR spectrum of bis-(cis-PE)-POSS

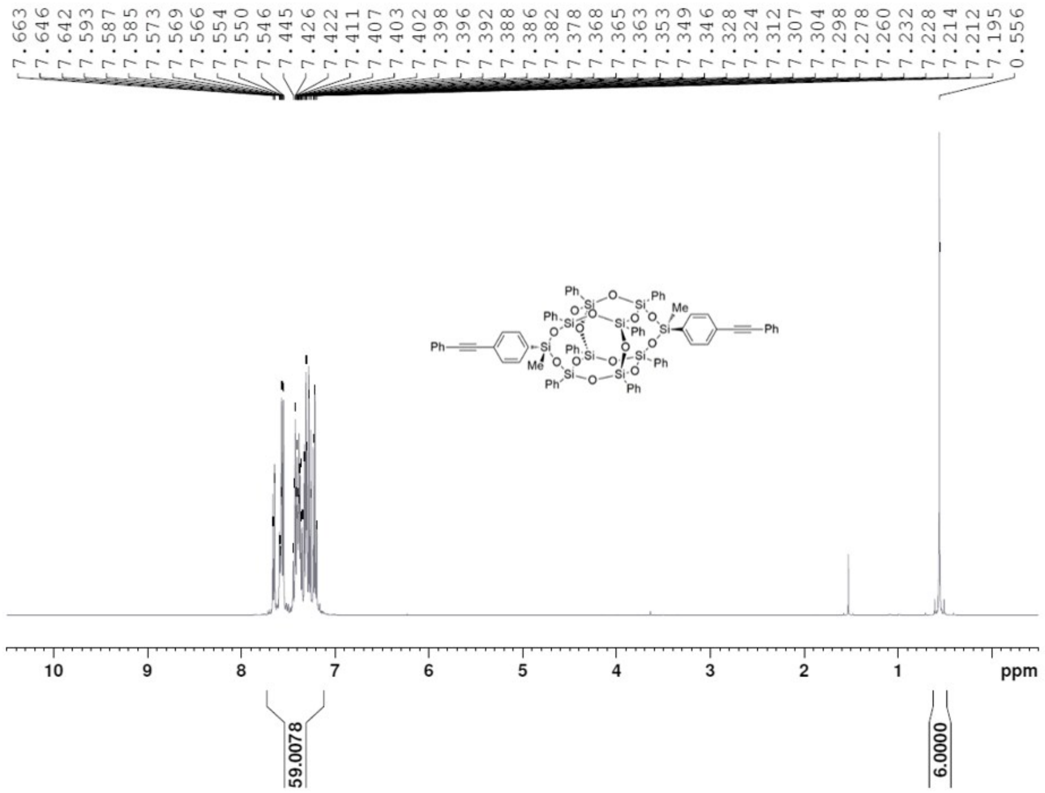

Figure S7. ^{1}H NMR spectrum of bis-(trans-PE)-POSS

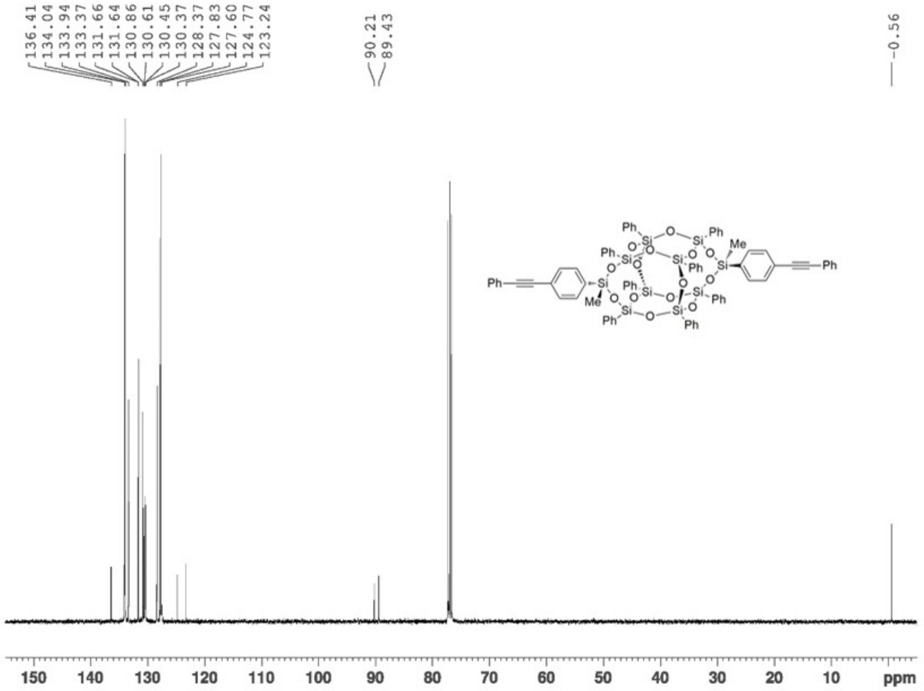

Figure S8. ^{13}C NMR spectrum of bis-(trans-PE)-POSS

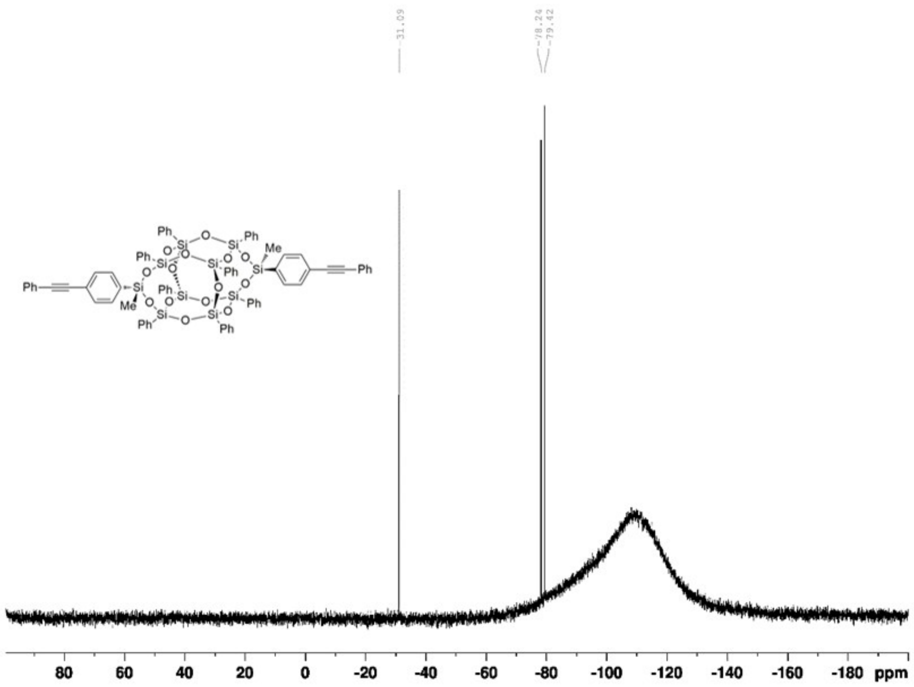

Figure S9. ^{29}Si NMR spectrum of bis-(trans-PE)-POSS

4. FTIR Spectra of bis-PE-POSS isomers

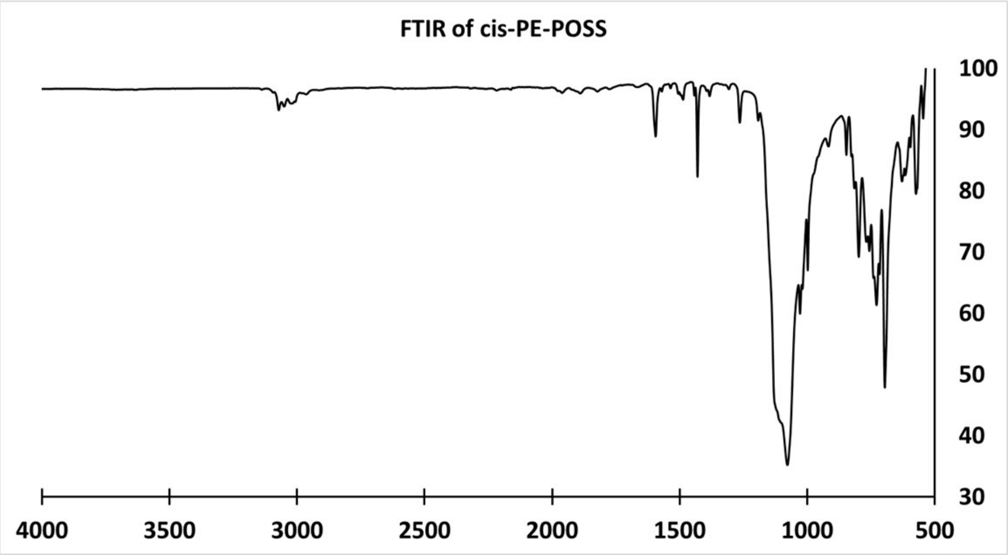

Figure S10. FTIR spectrum of bis-cis-PE-POSS.

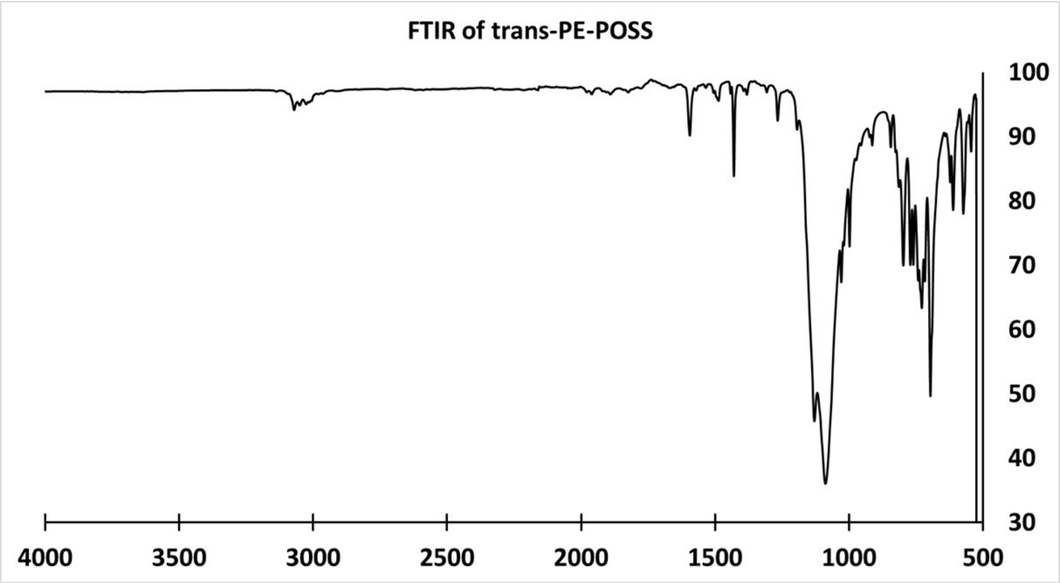

Figure S11. FTIR spectrum of bis-trans-PE-POSS.

5. Single-crystal X-ray diffraction analyses.

Crystals (either *cis* or *trans*) suitable for X-ray diffraction were grown from a solution of benzene in a 20 mL scintillation vial in air. The crystals, along with some of the mother liquor, were removed from the vessel via pipette and placed onto a microscope slide and immediately coated with a Fomblin® oil. A crystal was selected and mounted in the 90 K nitrogen cold stream provided by an Oxford Cryostream low-temperature apparatus on the goniometer head of a Bruker ApexII CCD instrument equipped with a copper fine-focus sealed tube (λ = 1.54178 Å). Data were collected employing omega and phi scans and reduced using Bruker SAINT.[1] Multiscan absorption correction was applied using SADABS.[1] Structure solution and refinement were conducted with SHELXS-2008[2] and SHELXL-2014,[3] respectively. All non-hydrogen atoms are refined anisotropically. Other refinement details are provided below and in the respective CIFs. Crystallographic data and structure details are reported below.

Structural analysis of cis-*(bis-PE)-POSS.*

The structure is found in the triclinic setting with space group $P\bar{1}$, $Z = 2$. The asymmetric unit is composed of one *cis*-(bis-phenylethynyl)-POSS molecule and two benzene molecules. One of the benzene molecules is disordered over two orientations with refined occupancies of 0.66(1):0.34(1). The remaining structural components are ordered. Figure S12 shows the molecular structure.

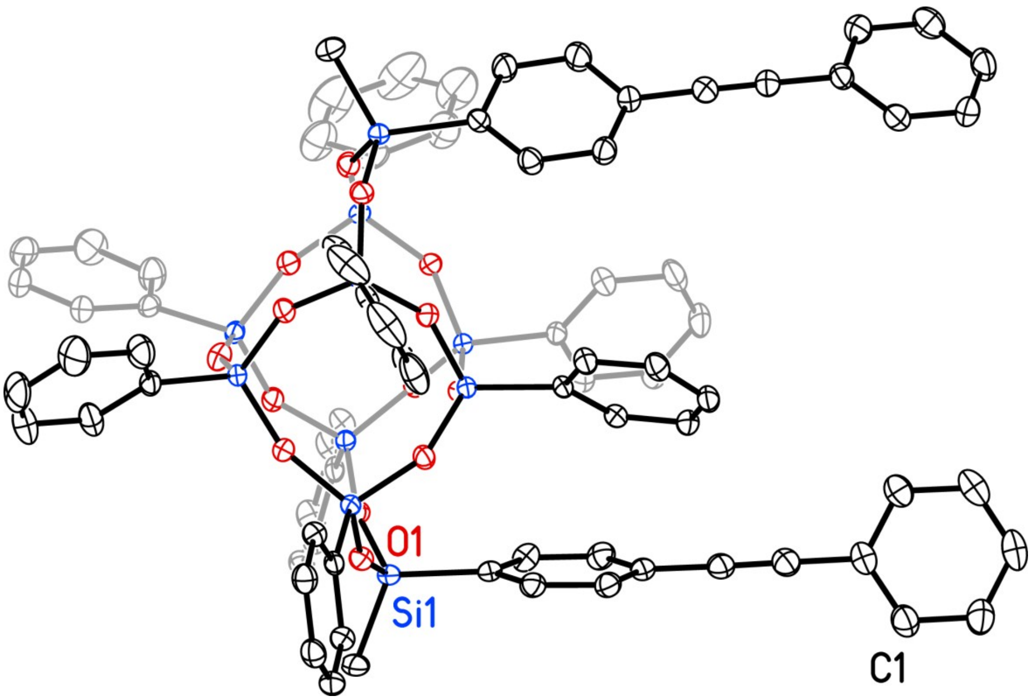

Figure S12. The molecular components of cis-bis-(phenylethynyl)-POSS drawn with 30% thermal contours. Hydrogen positions and solvent molecules are omitted for clarity.

Structural analysis of trans-*(bis-PE)-POSS.*

The structure is found in the triclinic setting with space group $P\bar{1}$, $Z = 1$. The asymmetric unit is composed of one half of the *trans*-(bis-phenylethynyl)-POSS molecule, three whole benzene molecules and one half benzene molecule. The POSS and half benzene molecules reside

on independent crystallographic centers of inversion. All structural components are fully ordered.

Figure S13 shows the molecular structure.

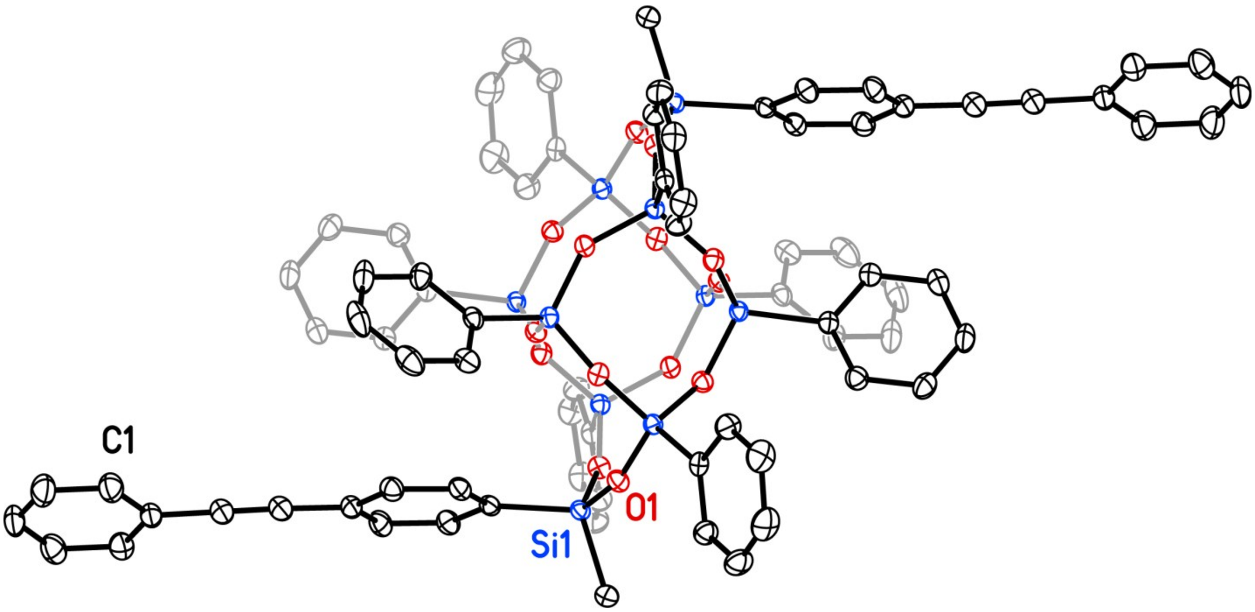

Figure S13. The molecular components of trans-bis-(phenylethynyl)-POSS drawn with 30% thermal contours. Hydrogen positions and solvent molecules are omitted for clarity.

Table S1. Crystallographic data of bis-PE POSS.

	***trans*-(bis-phenylethynyl)-POSS**	***cis*-(bis-phenylethynyl)-POSS**
chemical formula	$C_{120}H_{106}O_{14}Si_{10}$	$C_{90}H_{76}O_{14}Si_{10}$
formula weight	2052.94	1662.40
wavelength, λ (Å)	1.54178	1.54178
crystal system	triclinic	triclinic
space group	$P\bar{1}$	$P\bar{1}$
T (K)	90(2)	90(2)
a (Å)	10.7786(4)	13.9131(2)
b (Å)	15.4840(5)	14.5885(3)
c (Å)	18.0102(6)	23.0779(4)
α (deg)	109.5842(14)	97.0446(10)
β (deg)	97.1583(15)	102.9497(9)
γ (deg)	103.2582(18)	111.0906(8)
V (Å^3)	2689.10(16)	4151.42(13)
Z	1	2
d_{calc} (g cm^{-3})	1.268	1.330
μ (mm^{-1})	1.666	2.028
F(000)	1078	1736
crystal size (mm^3)	0.510 x 0.320 x 0.200	0.100 x 0.090 x 0.020
reflections collected	84678	115937
data / parameters / restraints	10474 / 650 / 0	15905 / 1078 / 0
R(int)	0.0395	0.0419
R_1 [$I > 2\sigma(I)$][a]	0.0351	0.0371
wR_2 (all data)[a]	0.0951	0.1022
Largest diff. peak and hole (e Å^{-3})	0.481 and –0.333	0.584 and –0.227

[a] $R_1 = \frac{\Sigma||Fo| - |Fc||}{\Sigma|Fo|}$; $wR_2 = \left\{\frac{\Sigma[w(Fo^2 - Fc^2)^2]}{\Sigma[w(Fo^2)^2]}\right\}^{1/2}$

6. Thermal Analysis

DSC data were recorded on a TA Instruments Q200 differential scanning calorimeter using a heat/cool/heat cycle at 10 °C/min from equilibration at 40 °C, to 315 °C, cooling to 40 °C, and heating to 450 °C. The first heat was used to determine the melting point of the compound, and the second heat to determine the cure exotherm.

Thermogravimetric analysis was performed on a TA Instruments TGA under nitrogen or air atmosphere. Samples were cured in-situ by first equilibrating at 40 °C and then ramping to 370 °C at 10 °C/min, followed by an isothermal hold at 370 °C for 60 minutes to cure the material. After the hold, the temperature was ramped again at 10 °C/min to 1000 °C. Onset of degradation was calculated to be the temperature at which 5% mass loss occurred, i.e., 95 % mass remaining.

7. Estimation of Hansen Solubility Parameters (HSP)

Following the methods reported earlier by Guenthner et al.,[4] a group contribution approach was utilized to compute both molar volumes and cohesive energies of the compounds, from which the solubility parameters are derived.[5] Using this approach, the parameter values for the cis and trans isomers, of necessity, will be equal. Such a result does not imply that the solubility characteristics will be the same, because the "radius of interaction" that defines the limits of solubility for the compounds may differ. The only constraint imposed by the assumption is that it should not be the case that some solvents are good solvents for the cis and poor solvents for the

trans, while others are good solvents for the trans and poor solvents for the cis. Experimentally, this is the case, as methanol is a poor solvent for both isomers, diethyl ether is a good solvent for the cis but not the trans isomer, and tetrahydrofuran is a good solvent for the cis isomer and a "less good" (but not necessarily poor) solvent for the trans isomer.

The tasks needed to estimate the HSP include 1) define chemical groups that facilitate estimation, 2) obtain cohesive energy and volume estimates for each group, then calculate the HSP 3) estimate the "radius of interaction", and 4) develop uncertainty estimates. These are discussed individually below.

Group Definition

This task is more important than it may seem superficially, as the choice of groups will determine which group contribution data is applicable. Because the data sources vary in reliability, the choice of groups will directly impact the reliability of the estimates themselves, and in fact the somewhat arbitrary nature of the choice provides a means for estimating uncertainty (discussed later).

Figure S14 illustrates the chosen grouping scheme. Although the silicate cage for thse compounds contains 10 Si and 14 O atoms, it can be divided into a T8 portion with two sets of additional Si ("D" type) and O atoms. These extra sets of atoms are identical to the backbone of a polysiloxane in atomic composition, which suggests that a polysiloxane grouping may be of use. In the chosen grouping scheme, the siloxane backbone is matched with a terminal methyl group and a terminal phenyl group to constitute a disconnected structure that is identical to the repeat unit of polymethyphenylsiloxane (PMPS). The remaining groups are the eight phenyl groups and two phenylethynyl groups.

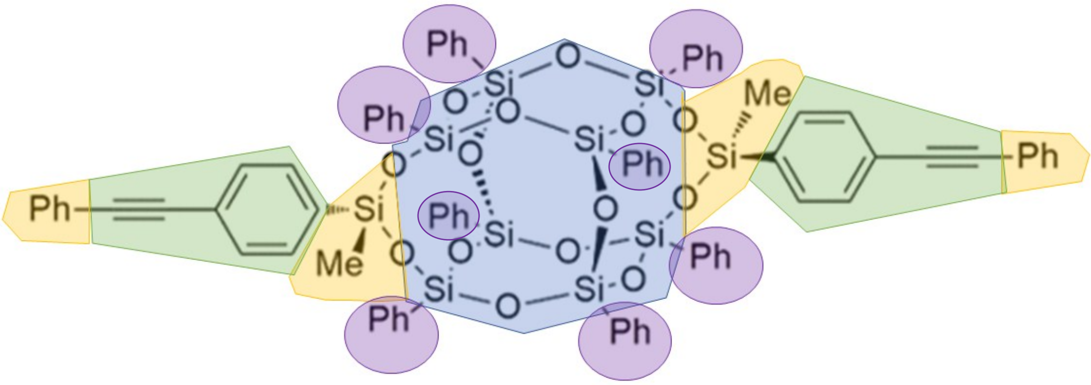

Figure S14. Group definition for the trans compound (the definition for the cis compound is identical), illustrating the T8 equivalent (blue, 1 per molecule, note how the two oxygen "half atoms" equate to a single oxygen), PMPS repeat unit equivalent (yellow, 2 per molecule), phenyl (purple, 8 per molecule), and phenylethynyl (green, 2 per molecule)

Determination of Group Contributions

Cohesive energy and volume components for the T8 cage and the phenyl group are available in Guenthner et al.[4] and shown in Table S2. For the PMPS repeat unit, a good estimate of the dispersion component of cohesive energy is available by interpolating the data of Humpa, Uhdeovl, and Roth,[6] which provides the total solubility parameter based on alkane interactions with chromatographic column packings. Given that in polysiloxanes, the polar and hydrogen bonding components are generally small, the total solubility parameter (especially one derived from alkane interactions) is likely to be quite similar to the dispersive component. To estimate this component, the tabular data of solubility parameter as a function of phenyl content in various PMPS materials from the reference was plotted and fit using a cubic polynomial, then interpolated to a phenyl fraction of 0.5, as shown in Figure S15. This procedure yielded an estimated solubility parameter (taken to be equal to the dispersive parameter δ_D) of 16.9 $(J/cc)^{1/2}$. The volume of PMPS was calculated based on the repeat unit molecular weight (136 g/mol) and density per the manufacturer (Aldrich) of 1.1 g/cc, resulting in 124 cc/mol, which in turn gives the dispersive cohesive energy component (E_D) as 35400 J/mol (see Table S2). To estimate the small polar and

hydrogen bonding contributions, we assumed that only the phenyl group contributes, so we simply set the E_P and E_H values equal to those of a phenyl group.

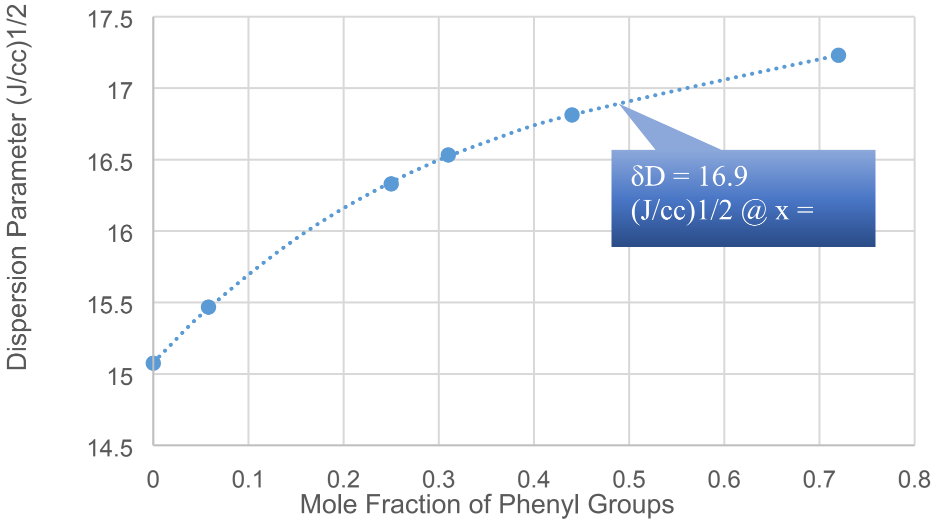

Figure S15. Determination of dispersive solubility parameter for PMPS by interpolation of tabular data from Reference 6.

For the phenylethynyl group, there is no readily available group data for cohesive energy density, although the correlation of Fedors[7] (often used in connection with cohesive energies) does provide a means of estimating the molar volume (one phenylene at 52.4 cc/mol + 2 non-hydrogenated *sp* carbons at 6.5 cc/mol each, for a total of 65.4 cc/mol). The correlation developed by Stefanis and Panayiotou[8] provides estimated HSP for the phenylethynyl group (see Table S3), which, together with the molar volume, provide a means of calculating the energy components (as shown in Table S2). Care must be taken when using this correlation for anything other than whole molecules because it was developed for liquid small molecules at ambient temperature, which causes it to be focused on molecules of a narrow size range. Because of its use of an extensive (group counting) method to calculate an intensive property, the correlation will fail if used for molecular fragments or repeating groups if these are too small (by providing solubility parameters

too close to the constant terms) or too large (by generating extreme values). Because the phenylethynyl fragment is similar in size to the kinds of whole molecules (e.g. ethylbenzene) used as liquid solvents under ambient conditions, the use of this correlation to calculate parameters for this fragment is unlikely to produce a major error.

Table S2. Summary of group contributions to HSP for phenylethynyl-containing POSS.

Frag-ment	V (cc/mol)	Source	δ_D (J/cc)$^{1/2}$	δ_P (J/cc)$^{1/2}$	δ_H (J/cc)$^{1/2}$	E_D (J/mol)	E_P (J/mol)	E_H (J/mol)	Source
T8	*134*	4	*22.0*	*18.9*	*15.0*	*65000*	*48000*	*30000*	4
Phenyl	*77.4*	7	19.7	0	2.2	*30000*	*0*	*360*	4
PMPS	*123.8*	MDS	*16.9*	0	1.7	35400	*0*	*360*	6*
PE	*65.4*	7	*19.2*	*6.3*	*4.5*	24100	2600	1300	8
Total**	1132	n/a	19.4	6.9	5.7	423900	53200	36200	n/a

Note: values in italics are those that are taken or calculated from an external source; the source numbers are equal to the reference numbers found in the list at the end of this document; PE = phenylethynyl, MDS = manufacturer's datasheet, * indicates source data has been re-analyzed and interpreted in this work, ** indicates that to obtain the total, the volume and energy components for 1 T8, 8 phenyl, 2 PMPS, and 2 PE groups are added together, the solubility parameters are then the square roots of the total energy components divided by the total volume

Table S3. Use of Stefanis and Panayiotou Correlation for Solubility Parameters of Phenylethynyl Fragment

Group Type	Times Used	Coefficient for δ_D (J/cc)$^{1/2}$	Coefficient for δ_P (J/cc)$^{1/2}$	Coefficient for δ_H (J/cc)$^{1/2}$
Constant	1	17.32	7.98	7.35
Aromatic CH	4	0.11	-0.53	-0.43
Aromatic C (no H)	2	0.84	0.61	0.01
Acetylene (no H)	1	-0.20	-0.76	-1.11
Total	n/a	19.2	6.3	4.5

Radius of Interaction

Table S4 provides the "radius of interaction" computed for the POSS compounds and the three solvents mentioned in this manuscript (diethyl ether, tetrahydrofuran, and methanol), and their characterization as a solvent or non-solvent (using anything greater than "sparingly soluble" as an

indication of solubility, even if the solubility was noted as worse in a relative sense). Because "some" trans compound dissolved in tetrahydrofuran, this instance was counted as soluble. For the cis compound, solubility exists at a radius of about 10 but not 19, so roughly the middle of this range, with a slight bias toward the lower end, or 12-16, represents a good estimate of the limit. For the trans compound, solubility exists at 6 but not at about 10, so 6-10 represents a good estimate of the limit. In both cases, the uncertainty associated with the radius is about two units, which is reasonable.

Table S4. Distance (in "energy difference" units[5]) between Phenylethynyl POSS and Solvents

Compound	δ_D (J/cc)$^{1/2}$	δ_P (J/cc)$^{1/2}$	δ_H (J/cc)$^{1/2}$	"Distance" (RED)
PE POSS	19.4	6.9	5.7	0
Tetrahydrofuran	16.8	5.7	8.0	5.8
Diethyl Ether	14.5	2.9	5.1	10.6
Methanol	15.1	12.3	22.3	19.4

Uncertainty Estimates for HSP

The main source of uncertainty in these estimates is not so much in the values themselves but in the assumptions that underlie their use. To estimate the uncertainty, we therefore undertook a sensitivity analysis to the assumptions, which involve mainly our choice of grouping and the data sources we selected. To do so, we constructed an alternate version of Table S2 (shown in Table S5), using a different set of assumptions and choices. For this alternate case, we chose to envision the entire silicate cage is a "120% version" of a T8 cage, since for the phenylethynyl POSS the cage has 24 atoms as compared to 20 for the T8 cage. We thus multiplied the energy and volume values from Guenthner et al.[4] by 120% to get the "cage contribution" and calculated HSP accordingly. For the peripheral groups, we assumed that there were 10 phenyl groups and 2 methyl groups per molecule, plus 2 phenylethynyl groups. For the phenyl and methyl groups, we assumed the volumes provided by Fedors.[7] For the methyl group, we assumed the total cohesive energy

given by Fedors was simply equal to the dispersive component, with all other components zero. For the phenyl group, we used the Stefanis and Panayiotou correlation[8] to obtain HSP, from which the energy components were calculated. For the phenyl ethynyl group, we calculated the values as before. This alternate approach was taken to be the "most different" case for which we felt we could attribute a confidence that the assumptions were not 'significantly worse" than our original choice. As outlined in Table S5, once the HSP for the POSS compound were calculated in this alternate way, we simply took the difference in each parameter from the original case as the characteristic uncertainty.

Table S5. Summary of group contributions (alternate approach for sensitivity analysis) to HSP for phenylene-ethynylene-containing POSS.

Frag-ment	V (cc/mol)	Source	δ_D (J/cc)$^{1/2}$	δ_P (J/cc)$^{1/2}$	δ_H (J/cc)$^{1/2}$	E_D (J/mol)	E_P (J/mol)	E_H (J/mol)	Source
T8*1.2	*161*	4	22.0	18.9	15.0	*78000*	*57600*	*36000*	4
Phenyl	*77.4*	7	*18.7*	*5.9*	*5.2*	27100	2700	2100	8
Methyl	*33.5*	7	5.8	0	0	*1125*	*0*	*0*	7
PE	*65.4*	7	*19.2*	*6.3*	*4.5*	24100	2600	1300	8
Total**	1132	n/a	18.8	8.9	7.3	392600	84900	56600	n/a

Note: values in italics are those that are taken or calculated from an external source; the source numbers are equal to the reference numbers found in the list at the end of this document; PE = phenylethynyl, ** indicates that to obtain the total, the volume and energy components for 1 T8*1.1, 10 phenyl, 2 methyl, and 2 PE groups are added together, the solubility parameters are then the square roots of the total energy components divided by the total volume

References

1. *SAINT* and *SADABS*; Bruker AXS Inc.: Madison, WI, **2014**.
2. *SHELXS*: Sheldrick, G. M. *Acta Crystallogr., Sect. A* **2008**, *64*, 112.
3. *SHELXL*: Sheldrick, G. M. *Acta Crystallogr., Sect. A: Found. Adv.* **2015**, *71*, 3.

4. Guenthner, A. J.; Lamison, K. R.; Lubin, L. M.; Haddad, T. S.; Mabry J. M. Hansen Solubility Parameters for Octahedral Oligomeric Silsesquioxanes. *Ind. Eng. Chem. Res.* **2012**, *51*, 12282-12293.
5. Hansen, C. M. *Hansen Solubility Parameters, A User's Handbook*, 2nd. Ed.; CRC Press: Boca Raton, FL, 2007.
6. Humpa, O.; Uhdeovl, J.; Roth, M. Thermodynamics of Poly(methylphenylsiloxane)-Alkane Systems by Gas-Liquid Chromatography. *Macromolecules* **1991**, *24*, 2514-2517.
7. Fedors, R. F. A Method for Estimating Both the Solubility Parameter and Molar Volumes of Liquids. *Polym. Eng. Sci.* **1974**, *14*, 147-154.
8. Stefanis, E.; Panayiotou, C. Prediction of Hansen Solubility Parameter with a New Gorup-Contribution Method. *Int. J. Thermophys*. **2008**, *29*, 568-585.
